# A new nanobody-enzyme fusion protein–linked immunoassay for detecting antibodies against influenza A virus in different species

**DOI:** 10.1016/j.jbc.2022.102709

**Published:** 2022-11-17

**Authors:** Pinpin Ji, Kun Wang, Lu Zhang, Zhenda Yan, Min Kong, Xuwen Sun, Qiang Zhang, Ning Zhou, Baoyuan Liu, En-Min Zhou, Yani Sun, Xinjie Wang, Qin Zhao

**Affiliations:** 1 Department of Preventive Veterinary Medicine, College of Veterinary Medicine, Northwest A&F University, Yangling, Shaanxi, China; 2 Shenzhen Branch, Guangdong Laboratory of Lingnan Modern Agriculture, Genome Analysis Laboratory of the Ministry of Agriculture and Rural Affairs, Agricultural Genomics Institute at Shenzhen, Chinese Academy of Agricultural Sciences, Shenzhen, China

**Keywords:** influenza A virus, antibodies against IAVs, nucleoprotein, nanobody, nanobody-HRP fusion protein, competitive ELISA, AIV, avian influenza virus, cELISA, competitive ELISA, HA, hemagglutinin, HEK-293T, human embryonic kidney 293T cell line, HI, hemagglutination inhibition, HRP, horseradish peroxidase, IAV, influenza A virus, IBV, influenza B virus, iELISA, indirect ELISA, IFA, immunofluorescence assay, IgG, immunoglobulin G, IV, influenza virus, M, membrane protein, mAb, monoclonal antibody, NA, neuraminidase, NDV, Newcastle disease virus, Ni, nickel, NP, nucleoprotein, NS, nonstructural protein, NTA, nitrilotriacetic acid, PBL, peripheral blood lymphocyte, PBST, PBS with Tween-20, PI, percentage inhibition, SPF, specific pathogen-free, TMB, tetramethylbenzidine, VHH, variable domain of the heavy chain of heavy-chain antibody

## Abstract

Circulation of influenza A virus (IAV), especially within poultry and pigs, continues to threaten public health. A simple and universal detecting method is important for monitoring IAV infection in different species. Recently, nanobodies, which show advantages of easy gene editing and low cost of production, are a promising novel diagnostic tool for the monitoring and control of global IAVs. In the present study, five nanobodies against the nucleoprotein of H9N2 IAV were screened from the immunized Bactrian camel by phage display and modified with horseradish peroxidase (HRP) tags. Out of which, we determined that H9N2-NP-Nb5-HRP can crossreact with different subtypes of IAVs, and this reaction is also blocked by positive sera for antibodies against different IAV subtypes. Epitope mapping showed that the nanobody-HRP fusion recognized a conserved conformational epitope in all subtypes of IAVs. Subsequently, we developed a nanobody-based competitive ELISA (cELISA) for detecting anti-IAV antibodies in different species. The optimized amount of coating antigen and dilutions of the fusion and testing sera were 100 ng/well, 1:4000, and 1:10, respectively. The time for operating the cELISA was approximately 35 min. The cELISA showed high sensitivity, specificity, reproducibility, and stability. In addition, we found that the cELISA and hemagglutination inhibition test showed a consistency of 100% and 87.91% for clinical and challenged chicken sera, respectively. Furthermore, the agreement rates were 90.4% and 85.7% between the cELISA and commercial IEDXX ELISA kit. Collectively, our developed nanobody-HRP fusion–based cELISA is an ideal method for monitoring IAV infection in different species.

Influenza is an acute infectious disease caused by the influenza virus (IV), which can rapidly evolve and can cause significant morbidity and mortality (1, 2). The IV, belonging to the Orthomyxoviridae family, is a negative-sense and single-stranded segmented RNA and an enveloped virus. The complete genome encodes RNA polymerase subunits, nucleoprotein (NP), matrix protein (M1), membrane protein (M2), nonstructural protein (NS1), nuclear export protein, hemagglutinin (HA), and neuraminidase (NA) (3, 4, 5). Based on the identity of the internal conserved proteins including NP and M1 proteins, the IVs are divided into types A, B, C, and D (6), of which, influenza A viruses (IAVs) are further divided into 18 H subtypes (H1–H18) and 11 N subtypes (N1–N11), according to their HA and NA makeup (7, 8). The highly variable IAVs are spread across a wide range of mammalian and avian species, such as humans, pigs, horses, dogs, cats, minks, seals, whales, and domestic and wild birds (4, 9). All subtypes of IAVs infect aquatic birds, which provide the genetic diversity required for the spread of IAVs across species (6, 10). Birds and pigs play important roles in global influenza pandemics caused by genomic reassortment and are the key hosts for the spread of influenza across the interspecies barrier (2, 11). Therefore, monitoring the history of IAV immunity and the infection of different susceptible species, especially in birds and pigs, is urgently needed for global IAV surveillance and control (12, 13).

Many serologic testing assays have been developed for detecting anti-IAV antibodies in the serum samples. These assays include hemagglutination inhibition (HI), agar gel immunodiffusion, virus neutralization test, immunofluorescence assay (IFA), and ELISA (5, 14). Of these, ELISA has advantages with high sensitivity, specificity, accuracy, and a high throughput and is thus widely used for early diagnosis and screening (12, 15). Since surface glycoproteins (HA and NA) of IAVs have a high rate of antigenic variability, it is problematic to develop a universal method for detecting antibodies against different subtypes of IAV using them as antigens (16). However, as we know, the NP is conserved among different subtypes of IAV. In addition, although the percentage of anti-NP antibodies is generally lower than the one of anti-HA antibodies, the antibodies against NP can also be quickly detected after IAV infected hosts. So, the NP was also widely used and one of the most promising diagnostic targets for IAV infection (17, 18).

There are indirect ELISA (iELISA) and competitive ELISA (cELISA), which use the highly conserved IAV-NP as an antigen to detect antibodies against different subtypes of IAVs in different species (19). However, most commercially available ELISA kits were produced based on the enzyme-conjugated second or monoclonal antibody (mAb). As the production of enzyme-labeled antibodies is complicated and shows a considerable degree of variation between lots, these kits are high in cost and make it difficult to control the consistency from batch to batch (20, 21).

Nanobodies, or named single-domain antibodies, are a novel type of engineered antibodies derived from the variable domain of the heavy chain of heavy-chain antibody (VHH) (22). Nanobodies have certain advantages, including their small molecular weight (15 kDa) and their suitability for genetic manipulation. Thus, they are widely used in disease diagnosis, surveillance, and prevention (22, 23). Regarding the development of disease diagnosis kits, derivatives can be easily established by coupling the reporters, and this is fiscally favorable compared with conventional antibodies (24). For example, the reporter-nanobody fusion protein (RANbody) has been applied in the development of detection assays and for labeling cells and tissues (25, 26). Recently, the nanobody-horseradish peroxidase (HRP) fusion was universally used to develop immunoassays to detect antibodies in the sera (20, 27). However, few studies have developed an immunoassay for detecting anti-IAV antibodies in the sera from different species using nanobodies as reagents.

In the present study, the NP from a subtype of the H9N2 avian IV (H9N2 AIV, H9N2-NP) was expressed using the *Escherichia coli* system. Then, H9N2-NP was used as an antigen to immunize the Bactrian camel and to screen the nanobodies obtained. In total, five nanobodies against H9N2-NP were obtained and modified to contain an HRP tag. Subsequently, the crossreactivity of these nanobodies with other subtypes of IAVs was assessed, and a nanobody recognizing a common epitope located in the NP of all IAV subtype strains was characterized. Then, using this nanobody-HRP fusion as a reagent, a cELISA was developed to qualitatively detect anti-IAV antibodies in the sera from different species. Our study provided a type of cELISA with rapid and easy operation for the diagnosis of IAV infection in different species. Importantly, the method produced was low in cost for following commercial production without the HRP-labeled antibodies *in vitro*.

## Results

### Expression and purification of the recombinant H9N2-NP

To immunize the camels, anti-H9N2-NP nanobodies were screened and the cELISA was established; the recombinant H9N2-NP was expressed using a bacterial system. SDS-PAGE analysis showed that the recombinant protein was successfully expressed and purified using a nickel–nitrilotriacetic acid (Ni–NTA) column with the expected size of 56 kDa (Fig. S1*A*). Western blotting analysis revealed that the recombinant H9N2-NP specifically reacted with positive chicken serum samples for anti-H9N2 AIV antibodies (Fig. S1*B*), indicating that the recombinant proteins possessed antigenicity.

### Construction of a phage display VHH library against the H9N2-NP

**Figure 1:**
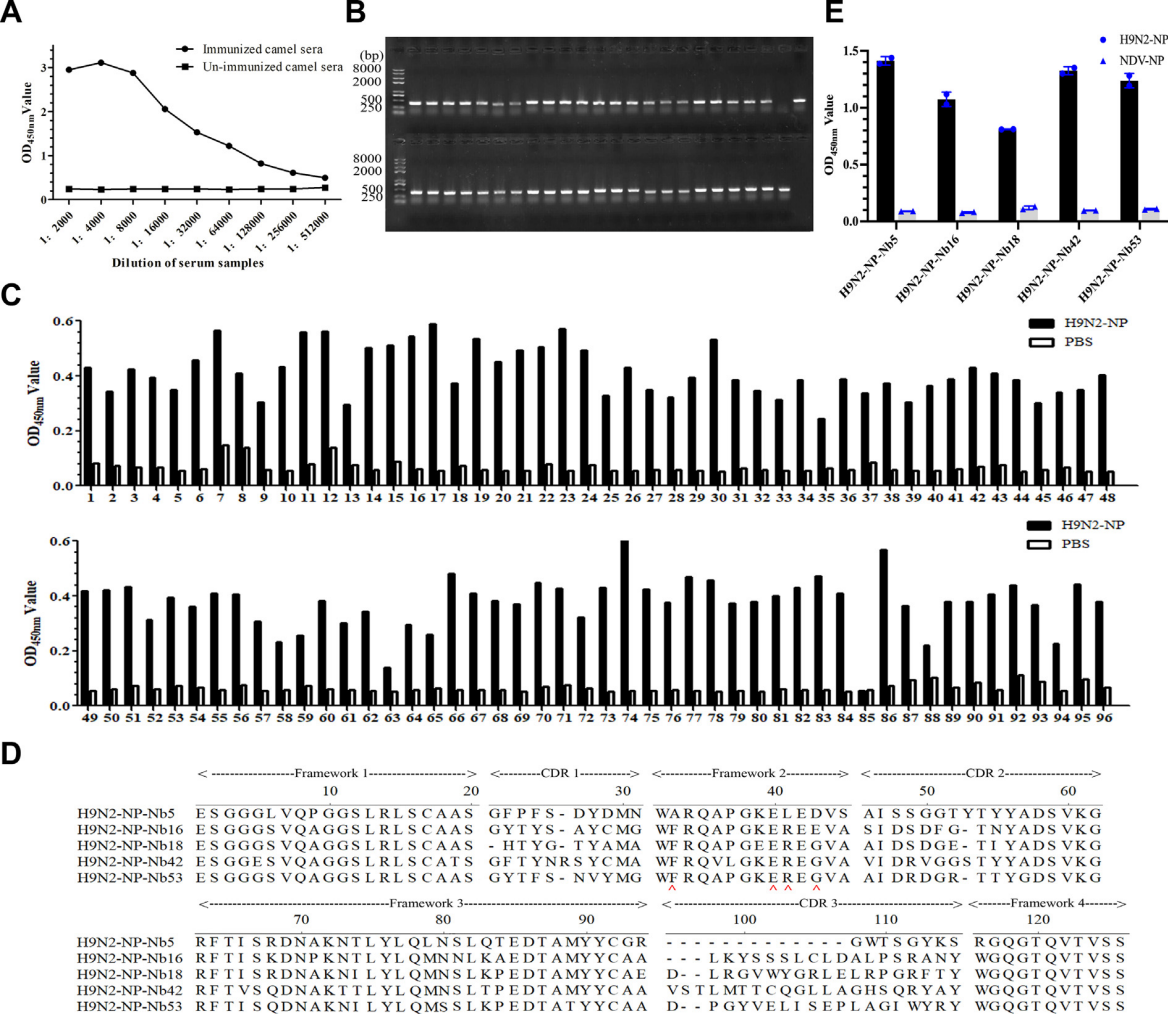
**Screening of the nanobodies against the recombinant H9N2-NP.***A*, titers of antibodies against H9N2-NP in the sera from the camel after the fifth immunization. *B*, identification of the positive rate for the VHH library by PCR. *C*, detection of the periplasmic extracts from 96 clones reacting with H9N2-NP by indirect ELISA. *D*, alignment of the amino acid sequences of the five screened nanobodies. Numbering and CDRs are in accordance with previous methods. *E*, determination of specific reactions between the five screened nanobodies and the H9N2-NP. CDR, complementarity-determining region; NP, nucleoprotein; VHH, variable domain of the heavy chain of heavy-chain antibody.

After the camel was immunized with the H9N2-NP, the titer of antibody against the protein in serum samples reached 1:128,000 based on iELISA (Fig. 1*A*). Next, a phage display library containing 9.8 × 10^8^ individual transformants was successfully constructed using the peripheral blood lymphocytes (PBLs) from the immunized camel. Subsequently, the results showed that the positive rate of the library was 98% by colony PCR assay (Fig. 1*B*), and each clone from the selected 96 clones contained a different VHH sequence (data not shown). The high capacity and positivity rate of the library confirmed the suitable heterogeneity of the library.

### Screening of nanobodies against H9N2-NP

After three rounds of panning, the results showed that the phage particles carrying H9N2-NP-specific VHH were significantly enriched (Table S2). Using the iELISA to detect the nanobodies in the periplasm from the 96 clones, the results showed that 94 clones could react with the H9N2-NP (Fig. 1*C*). These 94 positive clones were sequenced, and five nanobodies were obtained based on the complementarity-determining region 3 of the sequences. They were named H9N2-NP-Nb5, H9N2-NP-Nb16, H9N2-NP-Nb18, H9N2-NP-Nb42, and H9N2-NP-Nb53. Alignment results suggested that the five nanobodies possessed typical hydrophilic amino acid substitutions in the framework-2 regions (Fig. 1*D*). In addition, the five nanobodies were tested by iELISA using H9N2-NP and Newcastle disease virus (NDV)-NP separately as coating antigens. The results showed that they specifically reacted with the H9N2-NP (Fig. 1*E*) but not with NDV-VP, which was expressed with the same procedures and systems as H9N2-NP (27).

### Expression of the five nanobody-HRP fusion proteins against H9N2-NP

**Figure 2:**
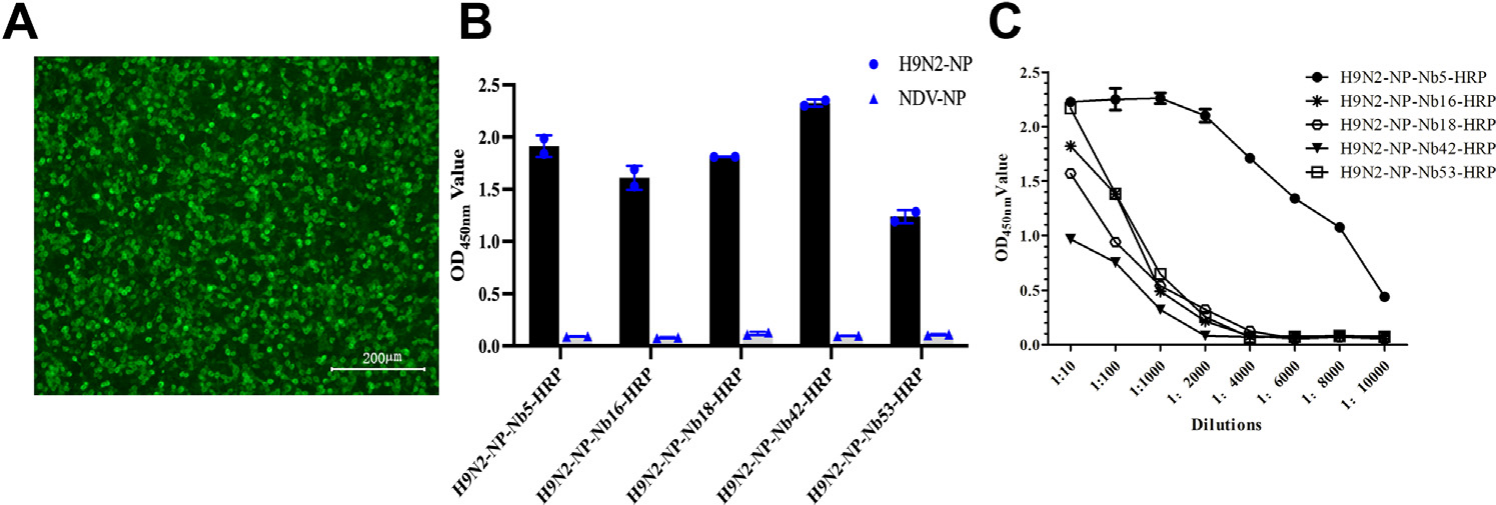
**Identification of five anti-H9N2-NP nanobodies with HRP fusion protein secretion expressed in HEK-293T cells.***A*, detection of the H9N2-NP-Nb5, H9N2-NP-Nb16, H9N2-NP-Nb18, H9N2-NP-Nb42, H9N2-NP-Nb53-HRP fusions expressed in HEK-293T cells by IFA. A representative image of H9N2-Nb5-HRP is shown, and similar results were observed for the other four nanobodies-HRP against H9N2-NP. *B*, analysis of the binding of H9N2-NP-Nb5, H9N2-NP-Nb16, H9N2-NP-Nb18, H9N2-NP-Nb42, and H9N2-NP-Nb53-HRP fusions with H9N2-NP by direct ELISA. *C*, titers of the H9N2-NP-Nb5, H9N2-NP-Nb16, H9N2-NP-Nb18, H9N2-NP-Nb42, H9N2-NP-Nb53-HRP fusions in the medium of HEK-293T cells using direct ELISA. HEK-293T, human embryonic kidney 293T cell line; HRP, horseradish peroxidase; IFA, immunofluorescence assay; NP, nucleoprotein.

Based on the aforementioned descriptions, the five anti-H9N2 nanobodies fusing with HRP were successfully expressed using human embryonic kidney 293T (HEK-293T) cells as confirmed by IFA identification (Fig. 2*A*). Using the supernatant from the transfected HEK-293T cells as testing antibodies, the results of direct ELISA showed that all five nanobody-HRP fusion proteins were secreted and subsequently termed H9N2-NP-Nb5-HRP, H9N2-NP-Nb16-HRP, H9N2-NP-Nb18-HRP, H9N2-NP-Nb42-HRP, and H9N2-NP-Nb53-HRP (Fig. 2*B*). The titer of the H9N2-NP-Nb5-HRP fusion proteins in the supernatants was 1:10,000, and the titers of the other four H9N2-NP-nanobodies-HRP fusions were 1:1000 (Fig. 2*C*).

### Crossreaction between nanobody-HRP fusion proteins and other IAV subtypes

**Figure 3:**
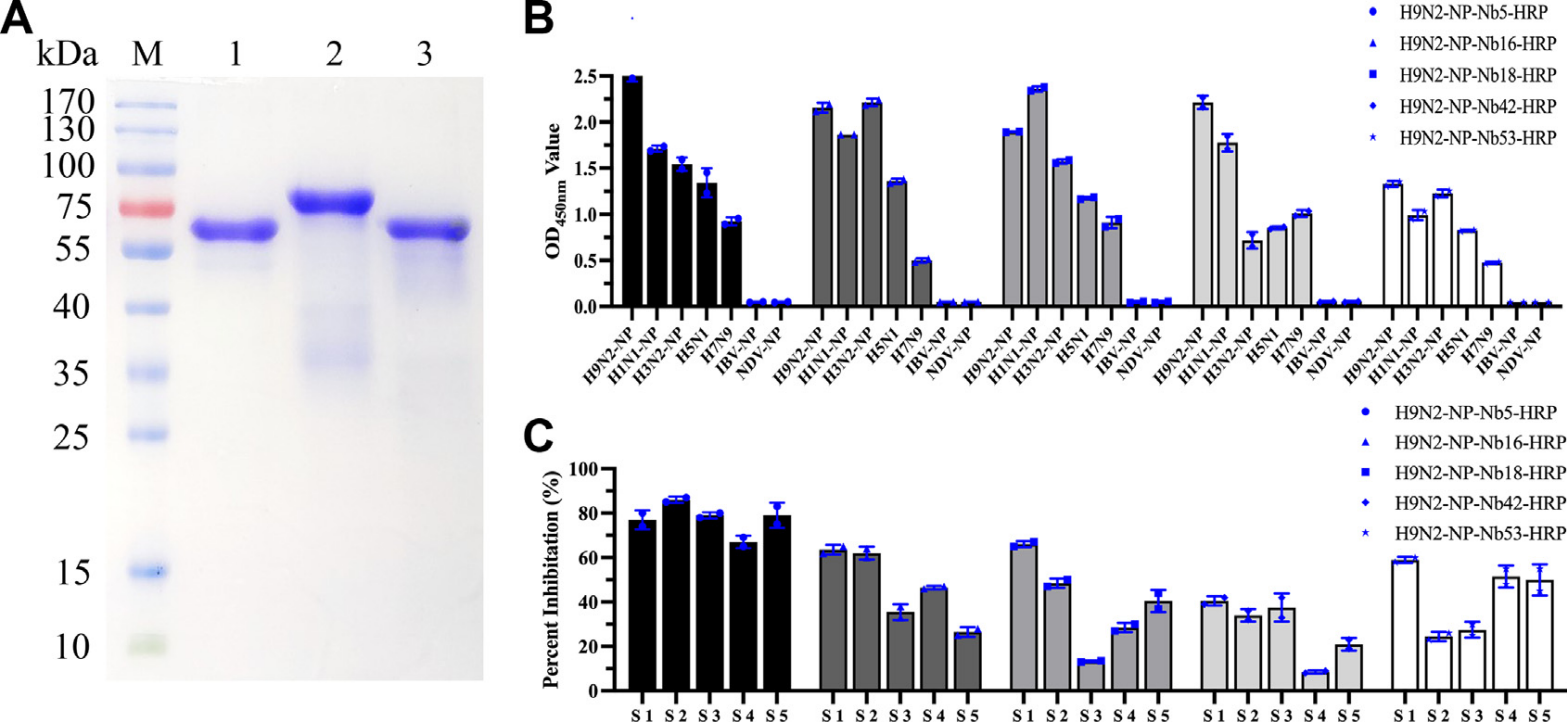
**Analysis of crossreactivity between the five nanobody-HRP fusion proteins and other subtypes of IAVs.***A*, SDS-PAGE analysis of H1N1-NP, H3N2-NP, and IBV-NP expressed using the *Escherichia coli* system. M, protein molecular markers; lane 1, H1N1-NP; lane 2, H3N2-NP, lane 3, IBV-NP. *B*, analysis of H9N2-NP-Nb5, H9N2-NP-Nb16, H9N2-NP-Nb18, H9N2-NP-Nb42, H9N2-NP-Nb53-HRP crossreacting with H1N1-NP, H3N2-NP, IBV-NP, and killed viral particles of H5N1 and H7N9 IAV by direct ELISA. *C*, analysis of the binding of five nanobody-HRP fusions to H9N2-NP blocked by the positive sera being separately for antibodies against H9N2 (S1), H1N1 (S2), H3N1 (S3), H5N1 (S4), and H7N9 (S5) using blocking ELISA. HRP, horseradish peroxidase; IAV, influenza A virus; IBV, influenza B virus; NP, nucleoprotein.

To analyze the five nanobody-HRP fusion protein crossreaction with other subtypes of IAV and influenza B virus (IBV), the direct ELISAs using the NP of H1N1, H3N2, B/Yamagata/16/1988, and killed denatured H5N1 and H7N9 particles as the coating antigens were performed. SDS-PAGE analysis showed that the NP of H1N1, H3N2, and B/Yamagata/16/1988 was successfully expressed using the *E. coli* system with the expected size and was successfully purified using a Ni–NTA column (Fig. 3*A*). The results of direct ELISA showed that the H9N2-Nb5, H9N2-Nb16, H9N2-Nb18, H9N2-Nb42, and H9N2-Nb53-HRP could crossreact with the NPs of subtypes H1N1, H3N2, H5N1, and H7N9 IAVs but not react with the NP of B/Yamagata/16/1988 (Fig. 3*B*). In addition, each positive serum sample for anti-H9N2, -H1N1, -H3N2, -H5N1, and -H7N9 antibodies confirmed using the commercial IDEXX ELISA kit was detected using these nanobody-HRP fusions as reagents by blocking ELISA. The results showed that the percentage inhibition (PI) of cELISA using H9N2-NP-Nb5-HRP as a probe reached 80% (Fig. 3*C*). The aforementioned results indicated that the epitope recognized by the H9N2-NP-Nb5 may be conserved across different subtypes of IAVs. Based on these results, H9N2-NP-Nb5-HRP was selected for identification of its recognizing epitope to analyze the conservation in all subtypes of IAVs and for developing the cELISA.

### Recognition of the conserved epitope recognized by H9N2-NP-Nb5-HRP among the different subtypes of IAV strains

**Figure 4:**
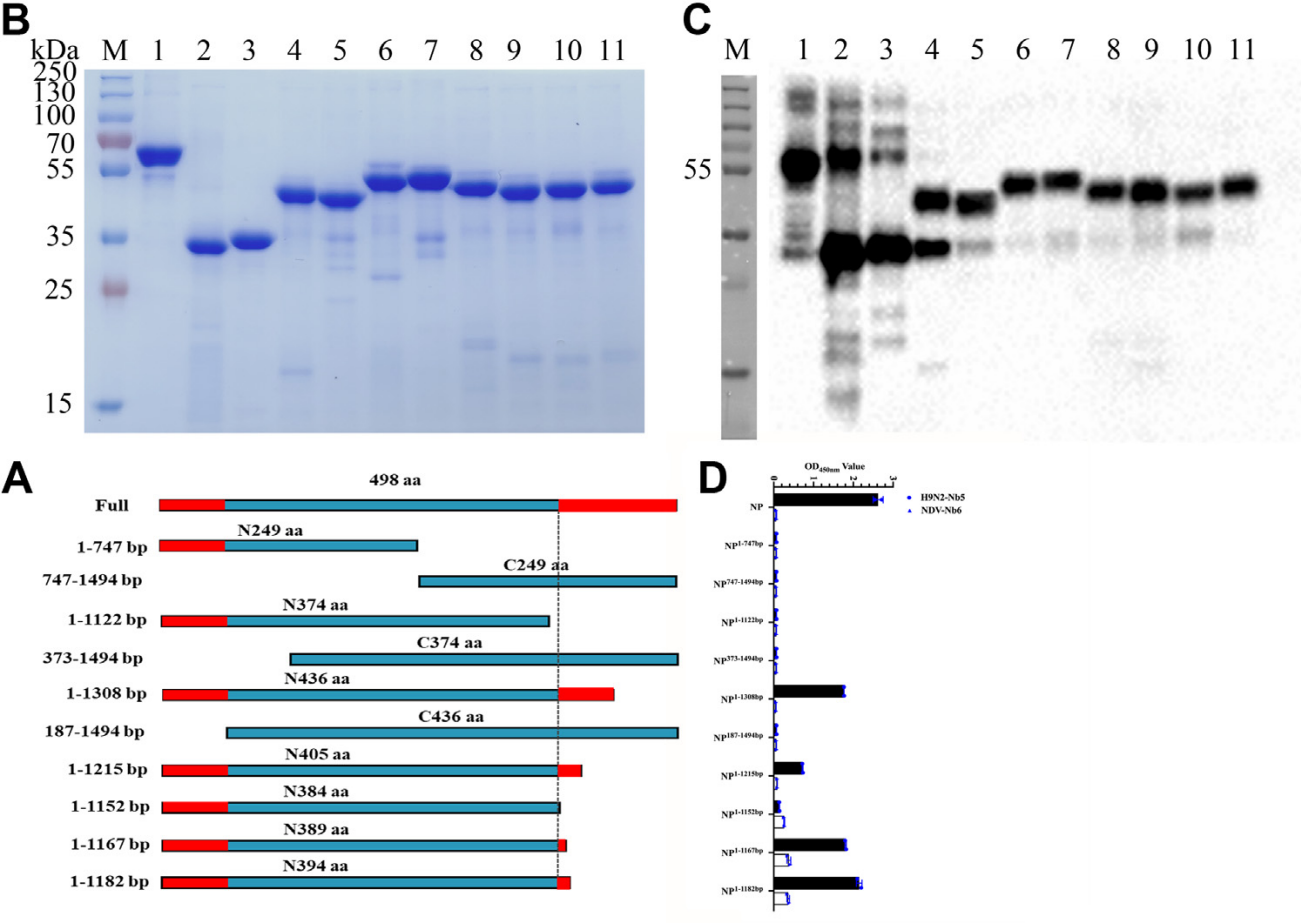
**Precise definition of the minimal motif recognized by H9N2-NP-Nb5-HRP.***A*, schematic diagram of different H9N2-NP truncated fragments. *B*, SDS-PAGE analysis of different fragments expressed using the *Escherichia coli* system. M, protein molecular markers; lane 1 to 11: full length of H9N2-NP, 1 to 747 bp (N249 amino acids), 747 to 1494 bp (C249 amino acids), 1 to 1122 (N374 amino acids), 373 to 1494 (C374 amino acids), 1 to 1308 bp (N436 amino acids), 187 to 1494 bp (C436 amino acids), 1 to 1215 bp (N405 amino acids), 1 to 1152 bp (N384 amino acids), 1 to 1167 bp (N389 amino acids), and 1 to 1182 bp (N394 amino acids). *C*, Western blotting analysis of expression of different fragments using anti-His monoclonal antibodies as the primary antibody. Lane 1 to 11: same as panel *B*. *D*, analysis of H9N2-NP-Nb5-HRP reacting with H9N2-NP and different truncated fragments of H9N2-NP by direct ELISA. HRP, horseradish peroxidase; NP, nucleoprotein.

To identify the epitope recognized by H9N2-NP-Nb5-HRP, different truncated and overlapping H9N2-NP fragments were designed (Fig. 4*A*). After these fragments were expressed using the *E. coli* system, SDS-PAGE and Western blotting analysis showed that they were successfully expressed with the expected size and purified by a Ni–NTA column (Fig. 4, *B* and *C*). The results of direct ELISA using these truncated fragments as coating antigens showed that the H9N2-NP-Nb5-HRP reacted with fragments spanning 1 to 1308 bp (N436 amino acids), 1 to 1215 bp (N405 amino acids), 1 to 1182 bp (N394 amino acids) and 1 to 1167 bp (N389 amino acids) but not with 1 to 747 bp (N249 amino acids), 747 to 1494 bp (C249 amino acids), 1 to 1122 (N374 amino acids), 373 to 1494 (C374 amino acids), 187 to 1494 bp (C436 amino acids), or 1 to 1152 bp (N384 amino acids) (Fig. 4*D*). These results suggested that the epitope recognized by H9N2-NP-Nb5-HRP may be a native conformational epitope and the two regions of amino acids 1 to 62 and amino acids 385 to 389 may be important domains for maintaining the conformation.

**Figure 5:**
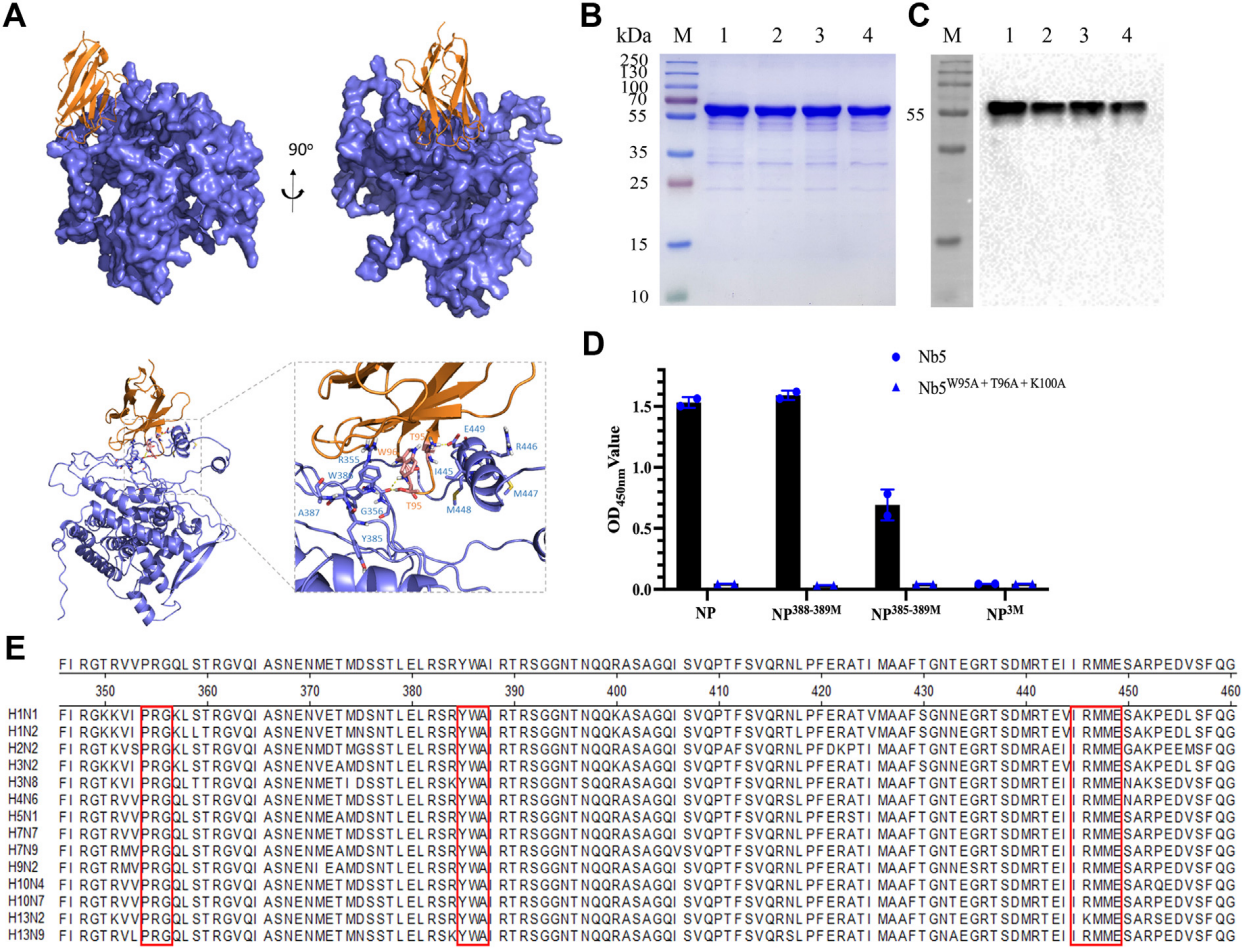
**Identification of conserved epitopes recognized by H9N2-NP-Nb5-HRP among the different subtypes of IAV strains.***A*, structure of the predicted docking complex between H9N2-NP-Nb5 and H9N2-NP. The H9N2-NP is shown in *blue* and H9N2-NP-Nb5 is shown in *brown*. *B*, SDS-PAGE analysis of different mutated H9N2-NPs expressed using the *Escherichia coli* system. M, protein molecular markers; lanes 1 to 4, H9N2-NP, H9N2-NP^388–389M^, H9N2-NP^385–389M^, and H9N2-NP^3M^. *C*, Western blotting to identify the expression of different mutated proteins with anti-His monoclonal antibodies. Lanes 1 to 11: same as panel *B*. *D*, determination of different mutated or wildtype H9N2-NP reaction with wildtype or mutated H9N2-NP-Nb5-HRP by direct ELISA. *E*, sequence alignments of the key motifs binding to H9N2-NP-Nb5 among different IAV strains. NP, nucleoprotein; HRP, horseradish peroxidase; IAV, influenza A virus.

To precisely define the epitope, AlphaFold2 server was used to predict the structure of proteins (28). The docking model showed that the W95, T96, and K100 are located in the H9N2-NP-Nb5 regions. Amino acids 354 to 356, 385 to 387, and 445 to 449 in the H9N2-NP were essential for H9N2-NP-Nb5-HRP interaction with the H9N2-NP (Fig. 5*A*). Based on the reaction of different fragments and predicted results, the amino acids located in 388 to 389 (H9N2-NP^388–389M^), 385 to 389 (H9N2-NP^385–389M^), and the three regions (H9N2-NP^3M^) predicted by the docking model were mutated into alanine (A) and expressed using the *E. coli* system. In addition, the W95, T96, and K100 of H9N2-NP-Nb5-HRP were also all mutated to A (termed H9N2-NP-Nb5^3M^-HRP). SDS-PAGE and Western blotting showed that the mutants of H9N2-NP were successfully expressed with the expected sizes (Fig. 5, *B* and *C*). Furthermore, the direct ELISA results showed that H9N2-NP^3M^ did not react with H9N2-NP-Nb5-HRP, and H9N2-NP-Nb5^3M^-HRP did not react with H9N2-NP. In addition, although H9N2-NP^385–389M^ still reacted with H9N2-NP-Nb5-HRP, the absorbance at 450 nm value of direct ELISA using the coated antigen was significantly lower than that of H9N2-NP (Fig. 5*D*). So, the results of direct ELISA using the mutated proteins indicated that the predicted amino acids for interaction between H9N2-NP and H9N2-NP-Nb5-HRP were correct. Furthermore, these results also suggested that the epitope recognized by the H9N2-NP-Nb5-HRP was a native conformational epitope.

To further analyze the amino acid conservation of the epitope, the amino acids 354 to 356, 385 to 387, and 445 to 449 of the NP from different IAV strains were aligned. Sequence alignments showed that the epitope was highly conserved among the different subtypes of IAV strains (Fig. 5*E*).

### Development of the cELISA using H9N2-NP-Nb5-HRP fusion protein as a probe

The conditions of cELISA using the H9N2-NP-Nb5-HRP fusion as the probe were optimized. Using checkerboard titration experiments with the direct ELISA, the results showed that the optimized amount of coated antigen was 100 ng/well and the optimal dilution of H9N2-NP-Nb5-HRP was 1:4000 (Table S3). Using the different dilutions of positive and negative sera for detection with the cELISA, the results showed that the optimized dilution of testing sera was 1:10 (Table S4). Finally, the results for determining the optimal times showed that the optimized incubation time between the mixture of H9N2-NP-Nb5-HRP with testing sera and coated antigen was 20 min and the optimal color reaction time was 10 min (Table S5).

### Cutoff value of the cELISA

The average PI (X) value of 180 negative sera from humans, pigs, and chickens detected by the developed cELISA was 1.0%, and the SD was 4.4%. The cutoff value of the cELISA was 14.2% (1.0% + 3 SD), indicating that the PI values of the tested sera by the cELISA were more than 14.2%, suggesting that the serum sample was positive for the anti-IAV antibody. If the PI value was less than 14.2%, this would then suggest they were negative.

### Sensitivity, specificity, reproducibility, and stability of the cELISA

**Figure 6:**
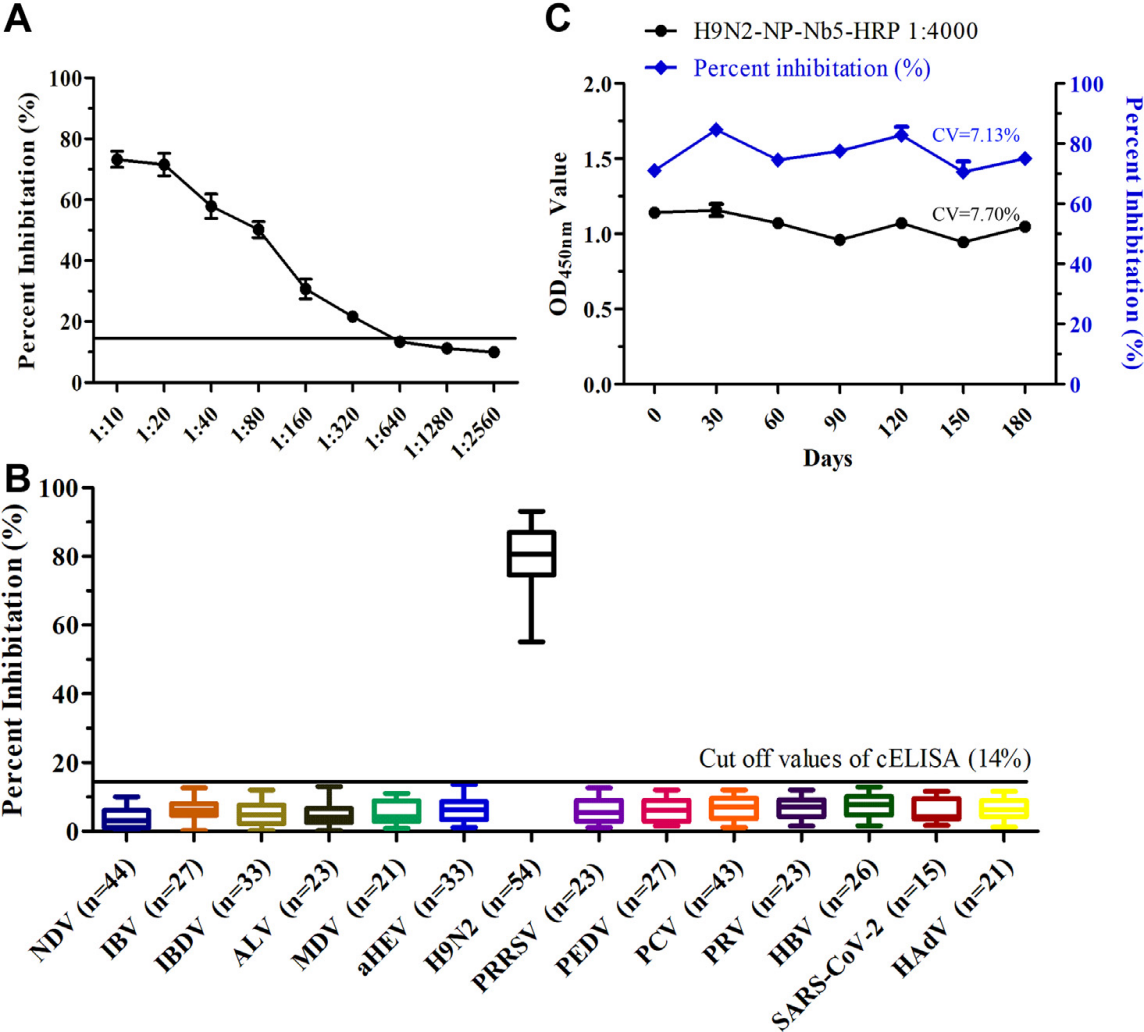
**Sensitivity, specificity, and stability of the nanobody-based cELISA for detecting anti-AIV antibodies.***A*, determination of the largest dilution of positive sera for anti-AIV antibodies. *B*, crossreaction of cELISA by detecting antibodies against antichicken, pig, and other human viruses. *C*, analysis of the stability of the cELISA. AIV, avian influenza virus; cELISA, competitive ELISA.

The results of testing different dilutions of positive sera for anti-IAV antibodies with the cELISA showed that the sera at a dilution of 1:640 was negative, and those at 1:320 were all positive (Fig. 6*A*). These indicated that the limits of detection of the cELISA for testing sera were 1:320.

The results of testing the positive sera for antichicken, pig, and human viruses’ antibodies showed that the PI values of these sera were all below 14.2%, suggesting that the specificity of cELISA was good (Fig. 6*B*).

To analyze the reproducibility of the cELISA, five positive and negative sera were tested to evaluate the intra-assay and interassay variabilities. The intra-assay CV of the PI was analyzed in the range of 3.14 to 7.02% with a median value of 5.08%, whereas the range for the interassay CV was 4.69 to 12.73% with a median value of 8.71% (Table S6). These data indicate that the cELISA method exhibited good reproducibility.

The results of direct ELISA using the plates and H9N2-NP-Nb5-HRP stored at 4 °C for 0, 30, 60, 90, 120, 150, and 180 days showed that the absorbance at 450 nm values were ∼1.0, indicating that the plates and the competitive agents H9N2-NP-Nb5-HRP remained active. The results of cELISA showed that the PI of cELISA stabilized at 80%, suggesting that the assay exhibited good stability over the past 180 days (Fig. 6*C*).

### Application of the cELISA to detect anti-IAV antibodies in the different species

To evaluate the assay for detecting anti-IAV antibodies in different species, the 2155 sera samples from human (n = 660), swine (n = 660), pet dog (n = 524), goat (n = 195), cattle (n = 28), rabbit (n = 44), cat (n = 44), pet dog (n = 524), duck (n = 98), and wild birds (n = 63) were simultaneously tested by the developed cELISA and the commercial IDEXX ELISA kit. For human sera, the positive rates of the cELISA and the commercial IDEXX ELISA kit were 97.58% (644 of 660) and 97.88% (646 of 660), respectively. For pig serum samples, the positive rates of the cELISA and the commercial IDEXX ELISA kit were 82.42% (544 of 660) and 83.33% (550 of 660), respectively. For pet dogs, the positive rates were 25.19% (132 of 524) and 25.95% (136 of 524), respectively. The positive rates of goat sera were 14.36% (28 of 195) and 14.87% (19 of 195), respectively. For cattle, the positive rates for both kits were 46.43% (13 of 28), and the positive rates of rabbit sera for both kits were 31.82% (14 of 44). For cat sera, the positive rates were 2.27% (1 of 44) and 6.82% (3 of 44), respectively. The positive rates of duck sera were 87.31% (227 of 260) and 88.08% (229 of 260), respectively, and for the wild birds, the rates were 27.73% (33 of 119) and 31.93% (38 of 119), respectively (Table 1). The aforementioned results indicated that the developed cELISA could be used to detect anti-IAV antibodies in different species.

**Table 1:** Detection of anti-IAV antibodies in the different species using the developed cELISA

Species	cELISA	Numbers of sera	Commercial IDEXX ELISA kit		Agreement (%)	Kappa value	Positive rate (%)
			+	−			
Human	+	644	644	0	99.7	0.93	97.6
	−	16	2	14			
Pig	+	544	544	0	99.1	0.97	82.4
	−	116	6	110			
Pet dog	+	132	131	1	98.9	0.97	25.2
	−	392	5	387			
Goat	+	28	26	2	97.4	0.90	14.4
	−	167	3	164			
Cattle	+	13	13	0	100	1.0	46.4
	−	15	0	15			
Rabbit	+	14	14	0	100	1.0	31.8
	−	30	0	30			
Cat	+	1	1	0	95.5	0.48	2.3
	−	43	2	41			
Duck	+	227	224	3	96.9	0.86	87.3
	−	33	5	28			
Wild bird	+	33	30	3	90.8	0.78	27.7
	−	86	8	78			

### Comparisons of the analytical performance among the cELISA, commercial ELISA kit, and HI test

A total of 185 clinical chicken sera and 91 sequential sera from the specific pathogen-free (SPF) chickens challenged with H9N2 AIV were tested by the cELISA, HI test, and commercial IDEXX ELISA kit. For the clinical chicken sera, the positive rates of the three assays were 57.30%, 57.30%, and 51.35%, respectively (Table 2). When the performance of three methods was compared, the cELISA allowed to identify 106 positive and 79 negative serum samples, whereas in the commercial IDEXX ELISA kit, only 90 were positive and 16 showed to be negative. When the 79 cELISA-negative samples were subjected to the commercial ELISA, 5 of them were positive and 74 were negative. The agreements were 90.4% between the cELISA and the commercial IDEXX ELISA kit (Table 2). The results of both cELISA and HI test coincided in 106 of the 185 serum samples with an agreement rate of 100%. In addition, the statistical analysis showed that there were no significant differences (*κ* values were >0.4) between the cELISA and either the HI test (*κ* values = 1.0) or the commercial IEDXX ELISA kit (*κ* values = 0.772). For the challenged chicken sera, the results showed that the positive rates were 73.63% (67 of 91), 61.54% (56 of 91), and 59.34% (54 of 91), respectively (Table 2). The agreements were 85.71% between the cELISA and the commercial IDEXX ELISA kit and 87.91% between the cELISA and HI test (Table 2).

**Table 2:** Comparison of the cELISA with HI test developed in the present study with the commercial ELISA using clinical samples and challenged chicken serum samples

Samples	cELISA	Numbers	Commercial IDEXX ELISA kit		Agreement (%)	Kappa value	HI test		Agreement (%)	Kappa value
			+	−			+	−		
Clinical chickens	+	106	90	16	90.4	0.77	106	0	100	1.0
	−	79	5	74			0	79		
Challenged chickens	+	67	54	13	85.7	0.69	56	11	87.9	0.73
	−	24	0	24			0	24		

**Figure 7:**
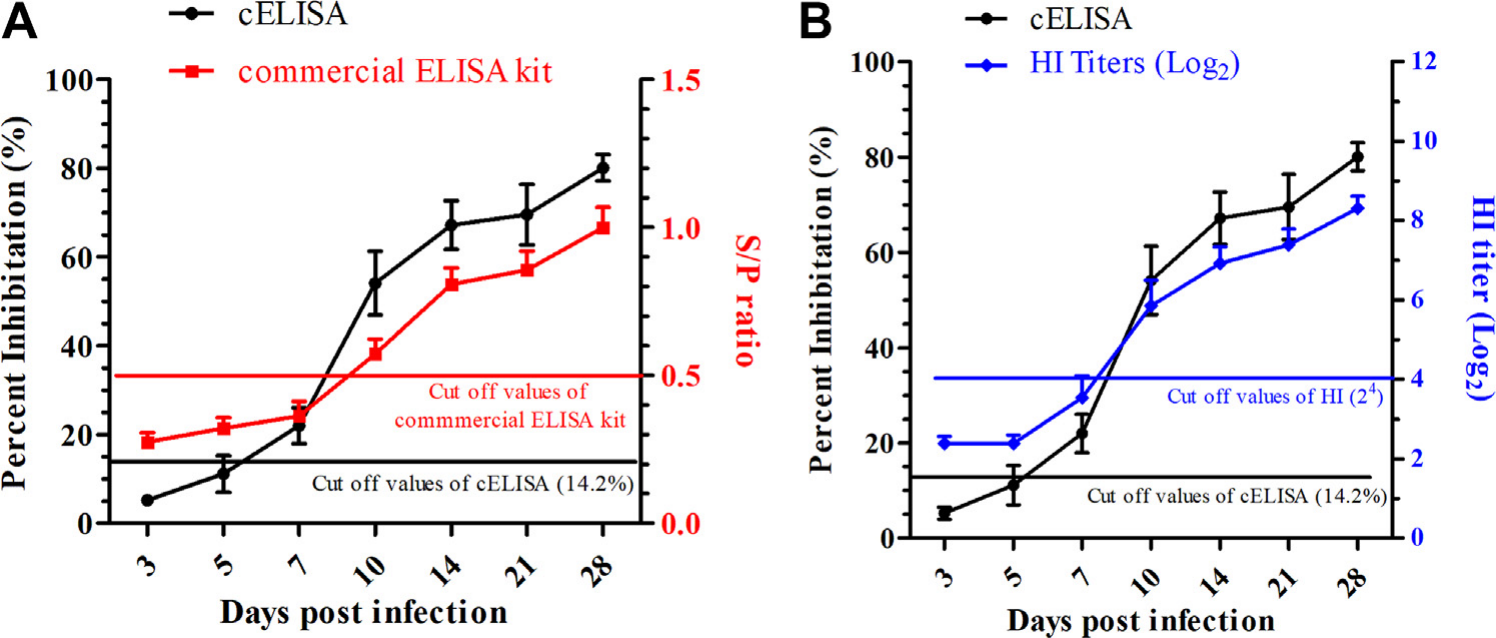
**Comparisons of the developed cELISA using an HI test and using a commercial ELISA kit by detecting sequential sera from the SPF chickens challenged with H9N2 AIV stock.***A*, detection of antibodies against H9N2 AIV in the serial sera from the challenged SPF chickens with the cELISA and a commercial IDEXX ELISA kit. *B*, detection of the above serial sera with the cELISA and HI assays. AIV, avian influenza virus; cELISA, competitive ELISA; HI, hemagglutination inhibition; SPF, specific pathogen-free.

In addition, the sensitivities of three approaches were also compared. All sera from 7 days postinfection were positive using cELISA, but only four samples were positive with the HI test and two samples were positive with the commercial IEDXX ELISA kit (Fig. 7*A*). At 5 days postinfection, one sample was positive using the cELISA, whereas all sera were negative with the HI test and commercial IDEXX ELISA kit (Fig. 7*B*). These results suggested that the developed cELISA was higher in sensitivity than the HI test and commercial IDEXX ELISA kit for detecting anti-IAV antibodies from challenged chicken sera.

## Discussion

Antibodies have proven to be central to the development of diagnostic methods, moving from polyclonal antibodies to the milestone development of mAbs. With the rapid development of mAb technology, many modern immunoassays have been developed and applied in a wide range of biological fields (29). Of these, ELISA has several advantages including high sensitivity, strong specificity, convenience, and fewer specific requirements with regard to experimental conditions and personnel, making it suitable for large-scale rapid screening of influenza immunity, and epidemic history (30). Competitive and blocking ELISAs to detect antibodies are useful methods for zoonotic diseases (or those that infect a wide range of hosts), as different secondary antibodies are required for the detection of antibodies from different species using iELISA. This is particularly true for the sera from certain rare species, for which the preparation of secondary antibodies is challenging (29). At present, most ELISA kits are developed using mAbs as detection reagents. Based on traditional antibodies, although the competitive and blocking ELISAs avoid the use of secondary antibodies against different species, they require *in vitro* labeling of mAbs, resulting in poor batch-to-batch consistency (31). In addition, the large size of mAbs makes them a challenging substrate for recombinant gene editing, and the high cost of production technology limits their development to some extent (32). Nanobodies are relatively novel diagnostic probes with lower molecular weights than traditional mAbs, with *in vitro* recombination and protein expression methods using bacterial production systems (33). In addition, the simpler gene structure of nanobodies allows for easier modification using fusion constructs with reporter genes, which greatly simplifies the commercial production of ELISA diagnostic kits. In this study, based on these advantages, five anti-H9N2 nanobodies were screened, and a nanobody-HRP fusion protein recognizing the common epitope of all subtypes of IAV strains was selected to establish a cELISA for detecting anti-IAV antibodies in the different species. The developed cELISA based on the nanobody fusion HRP protein not only overcame the aforementioned shortcomings but also showed good sensitivity, specificity, and agreement with a commercial ELISA kit. The developed assay utilized the advantages of nanobody fusion with HRP, which simplified the operational steps and shortened the detection time (∼40 min in total). More importantly, the diagnostic kit could be assembled by directly collecting the culture supernatant of the cell line stably expressing the nanobody-HRP fusion protein without antibody labeling, alleviating the need for the complex preparation process of species-specific secondary antibodies and inconsistency between batches, whilst also reducing the cost of commercial production. The developed cELISA could detect anti-IAV antibodies in the serum samples of different species, with a high coincidence rate and no statistically significant difference compared with existing commercial ELISA kits. There was also no statistically significant difference for the developed cELISA and HI test. Compared with the commercial ELISA kits and HI test for detection of anti-IAV antibodies in different species, the method developed in the present study had obvious advantages regarding a simpler preparation process, lower cost, higher sensitivity, and shorter detection time, and thus has the potential to become a technology for the detection of IAVs in the future. In addition, the platform of developing the cELISA based on the nanobody-HRP fusion is a novel and promising strategy for designing diagnostic kits for zoonotic disease, especially for pathogens with a wide range of potentially infectable hosts.

The IV is a serious threat to human life and has caused global public health concerns (3). As IAVs can infect a series of hosts, including humans, poultry, pigs, minks, dogs, and other mammals, especially deep-sea mammals (6), the development of a universal method for diagnosis in different species is important. The results of this study showed that the established cELISA could detect anti-IAV antibodies in humans, pigs, poultry, dogs, cattle, sheep, and other species. In addition, we also found that the epitope recognized by H9N2-NP-Nb5-HRP is highly conserved among all the different subtypes of IAVs. Therefore, we speculate that this method can be applied to the detection of anti-IAV antibodies in all species. Of course, in future studies, the more sera from different species will be assessed to verify the universality of the assay.

It has been reported that AIV infections exist in the host of wild birds, resulting in the continuous spread of avian influenza across the world. Therefore, closer monitoring of AIV in wild birds is very important for better understanding the scope and transmission of infection (34). In the present study, the results showed that the established cELISA could detect anti-IAV antibodies in the sera from wild birds. As the number of wild birds used in the study was small, we will continue to collect the sera of different wild birds to prove the feasibility of the assay in detecting antibodies against AIV in wild birds. However, based on these data, it is hypothesized that the cELISA is a suitable means for monitoring the AIV infection in the wild bird flocks.

To date, six epitopes have been determined in the NP from H5N1, H3N3, and H1N1 subtype IAVs (35, 36). However, all the aforeidentified epitopes were linear. In this study, we identified a conformational epitope of NP recognized by H9N2-NP-Nb5 and found that it is highly conserved in different subtypes of IAV. The epitope was predicted using the AlphaFold2 server, and certain regions for the reaction between H9N2-NP-Nb5-HRP and H9N2-NP were also determined using the different truncated and mutated fragments. However, the key amino acids have not been identified yet. In future studies, cryo-electron microscopy should be used to determine the spatial conformation of the antigen–nanobody complex and identify the key motifs. In addition, our study showed that the epitope could induce strong immune responses in the different species after natural IAV infection. This suggested that the common epitope may serve an important function, which will be evaluated in future studies.

In conclusion, a common and highly conserved epitope in the NP of different subtype IAVs recognized by the H9N2-NP-Nb5 was identified. Next, a cELISA using H9N2-NP-Nb5-HRP as a probe was developed, and it exhibited good sensitivity, specificity, and high consistency with the commercial IDEXX ELISA kit. Importantly, the advantages of the developed cELISA over commercial kits include the elimination of enzyme-labeled antibodies *in vitro*, consistency between batches, and a simpler preparation process, lower cost, higher sensitivity and specificity, and shorter detection time (∼40 min in total). The developed cELISA was a suitable method for surveillance and monitoring of IAV infection in different species.

## Experimental procedures

### Cells and viruses

HEK-293T cells were obtained from the American Type Culture Collection and cultured in Dulbecco’s modified Eagle’s medium (Thermo Fisher Scientific, Inc) supplemented with 10% fetal bovine serum (Gibco; Thermo Fisher Scientific, Inc) at 37 °C in a humidified incubator supplied with 5% CO_2_.

A/chicken/Hebei/LC/2008 (H9N2 AIV, HB08 strain) was propagated in the allantoic cavity of 9-day-old chicken embryonated eggs. The inactivated and purified H5N1 and H7N9 viral particles were kindly provided by Harbin Veterinary Research Institute, Chinese Academy of Agricultural Sciences.

### Serum samples

The 180 negative sera from humans (n = 65), pigs (n = 60), and SPF chickens (Beijing Merial Vital Laboratory Animal Technology Co, Ltd; n = 55) were used to calculate the PI of the developed cELISA. All these serum samples were confirmed using an IDEXX AIV antibody ELISA kit IDEXX and HI assay. The commercial ELISA kit is the most stable and used in laboratory tests at present (20, 37). And the HI assay is the serological “gold standard” method specified by OIE (38, 39).

In addition, to evaluate the agreements of the developed cELISA with the IDEXX commercial ELISA kit and with the HI assay, 91 sera collected from 13 SPF chickens challenged with H9N2 allantoic fluid (HA titer: 2^9^) and 185 clinical sera were used for the simultaneous detection of three methods (27). To determine the crossreactivity of the cELISA, cELISA was used to assess the 181 clinical positive chicken sera against other avian viruses, including NDV (n = 44), avian infectious bronchitis virus (n = 27), infectious bursal disease virus (n = 33), avian leukemia virus (n = 23), Marek’s disease virus (n = 21), and avian hepatitis E virus (n = 33), to assess the 116 clinical positive pig sera against other viruses, including porcine reproductive and respiratory syndrome virus (n = 23), porcine epidemic diarrhea virus (n = 27), porcine circovirus type 2 (n = 43), and pseudorabies virus (n = 23), and to assess the 69 positive human sera against other human viruses including hepatitis B virus (n = 26), severe acute respiratory syndrome coronavirus 2 (n = 15), and human adenovirus (n = 21).

### Expression and purification of the recombinant H9N2-NP

The H9N2 AIV strain HB08 was used as the template for amplification of the viral gene encoding the NP by RT–PCR. The sequences of the primers used for PCR amplification are listed in Table S1. The gene was cloned into the pET-28a vector (Novagen) using the EcoRI and XhoI restriction enzyme sites. To express the recombinant H9N2-NP, the recombinant positive plasmids were transformed into *E. coli* Transetta (DE3) cells (TransGen Biotech). Then, 0.5 mM IPTG was added for inducing the expression of recombinant H9N2-NP at 25 °C for 12 h. After centrifugation at 8000*g* for 15 min at 4 °C, the bacteria were collected. After the bacterial cells were sonicated, the recombinant protein was purified using an Ni–NTA column (GE Healthcare). Expression, purification, and antigenicity of the recombinant protein were analyzed by SDS-PAGE and Western blotting.

### Bactrian camel immunization and library construction

A 4-year-old male Bactrian camel was immunized with the purified recombinant H9N2-NP (1 mg/ml) with an equal volume of complete Freund’s adjuvant (Sigma–Aldrich; Merck KGaA) subcutaneously (20, 26). Five consecutive immunizations were performed every 2 weeks. For the subsequent four administrations, the recombinant proteins were emulsified with incomplete Freund's adjuvant, and the injection volume was the same as that used for the first time. After the last immunization, the sera were collected from the immunized camel and used to test the titer of anti-H9N2-NP antibodies with iELISA using purified H9N2-NP as a coating antigen.

A total of 5 days after the last immunization, 200 ml of peripheral blood was collected from the immunized Bactrian camel and diluted with an equal volume of RPMI1640 media (01-100-1ACS; Biological Industries). Next, the Ficoll-Paque PLUS Lymphocyte Separation Fluid (Greiner Bio-One) was used to isolate PBLs according to the manufacturer’s protocol. Total RNA was extracted from the PBLs and used as templates for reverse transcription to synthesize the complementary DNA. Next, the VHH genes were amplified using nested PCR as described previously (27, 40, 41). The sequences of the primers used for nested PCR are listed in Table 1. Subsequently, the VHH genes were cloned into a phage display pMECS vector using the PstI and NotI restriction enzyme sites. The recombinant “phagemids” were electroporated into 1 ml fresh *E. coli* TG1-competent cells. The cells were plated on LB agar plates supplemented with ampicillin and d-glucose at 37 °C. The bacterial monolayer was scraped and added to LB containing 20% glycerin and stored at −80 °C. Then, the positive rate and diversity of the library were determined as described previously (42).

### Screening of anti-H9N2-NP–specific nanobodies

To screen the specific nanobodies against the H9N2-NP, three rounds of panning were performed as previously described (40, 41). Briefly, the 96-well plates (Nunc, Thermo Fisher Scientific, Inc) were coated with the purified recombinant H9N2-NP (5 μg/well) using PBS (pH 7.2) as the coating buffer. Then, the blocking buffer (2.5% skimmed milk in PBS, 300 μl/well) was added to the plates at 37 °C for 2 h. After washing with PBST (1 l PBS with 1 ml Tween-20, 300 μl/well), the recombinant phages rescued *via* M13K07 (5 × 10^11^ plaque-forming unit/ml) were added and incubated for 2 h at room temperature. Then, the binding phages were eluted with fresh trimethylamine (100 mM) and neutralized with Tris–HCl buffer (1 M, pH 7.4). The titers of eluted phages were tested by infecting TG1 cells and then used for the next round of rescue and panning. After three rounds of screening, the ratio of the output of the positive phage to the output of the negative phage was calculated to evaluate the enrichment of phages against H9N2-NP. A total of 96 clones were selected and induced by 1 mM IPTG for expression of anti-H9N2-NP nanobodies. The soluble nanobodies were extracted from the periplasm and tested by iELISA using the H9N2-NP as the coating antigen. The NDV-NP was used as a negative control for the iELISA. The protein was also expressed using the same vector pET-28a described in a previous study (27). Finally, all VHH genes from the positive clones were sequenced (Tsingke Biotechnology Co, Ltd) and classified according to their complementarity-determining regions of the amino acid sequence.

## iELISA

For determining the titers of immunized Bactrian camel and specific detection of the periplasm containing the nanobodies, iELISA was performed based on a previous study (41). Briefly, the 96-well plates were coated with the purified H9N2-NP (400 ng/well) overnight at 4 °C and then were blocked with blocking buffer at 37 °C for 1 h. After the plates were washed three times with PBST, the sera from the immunized camel or the periplasm were added to the wells and incubated at room temperature for 1 h. After washing three times, for titration of camel sera, the rabbit anticamel antibody and HRP-labeled goat anti-rabbit immunoglobulin G (IgG) (TransGen Biotech) were added sequentially. For testing the periplasm, the anti-HA-tag antibody (GenScript Biotech Corp) and HRP-labeled goat antimouse IgG (TransGen Biotech) were subsequently added. After washing again as aforementioned, tetramethylbenzidine (TMB) was added to the plates, and the plates were incubated at room temperature for 15 min to allow the development of the color. Subsequently, the reaction was halted by the addition of 3 M H_2_SO_4_, and the absorbance at 450 nm value was read using an automatic ELISA plate reader (Bio-Rad Laboratories, Inc).

### Expression of the anti-H9N2-NP nanobody with HRP fusion proteins

The nanobody-HRP fusion protein was expressed in HEK-293T cells as described previously (43). Briefly, the VHH genes encoding the nanobody were obtained from the positive pMECS plasmids by digesting with PstI and NotI enzymes and ligating into the vector pCMV-N1-HRP, which was also digested with the same two enzymes, as described previously (27). After the positive recombinant plasmids were identified by sequencing, they were transfected into the HEK-293T cells with polyetherimide (Polysciences, Inc) agent. A total of 48 h post-transfection, the culture supernatant containing the nanobody-HRP fusion proteins were collected and supplemented with 0.02% w/v NaN_3_. For identification of expression, the transfected cells were analyzed using an indirect IFA with anti-His mAbs as the primary antibody and FITC-labeled goat antimice IgG antibodies as the secondary antibody. Direct ELISA was performed to quantify the secreted expression of nanobody-HRP fusion proteins in the HEK-293T cells as described previously (27). Briefly, after the plate had been coated with the purified H9N2-NP, the culture medium (100 μl/well) was added and incubated for 1 h at room temperature. As aforementioned, the TMB color reactions were halted with 3 M H_2_SO_4_, and absorbance at 450 nm value was obtained.

### Crossreaction of the nanobody-HRP fusion protein with other IAV subtypes

Cross-reactions of these nanobody-HRP fusion proteins with other subtypes of IAVs were also assessed using the direct ELISA described previously. The A/Guangzhou/01/2009 (H1N1), A/swine/Guangxi/1659/2017 (H3N2), H5N1 (Re-12), H7N9 (H7-Re3) IAV strain, and IBV strain (B/Yamagata/16/1988) were selected for use as coating antigens. Of these, the H1N1 and H3N2 IAVs NPs and IBV NP were also expressed using the bacterial system as for the H9N2 NP and used as coating antigens in the direct ELISA. For H5N1 and H7N9, the purified and denatured viral particles were used as coating antigens.

In addition, to further analyze crossreaction, the blocking rate of the nanobody-HRP fusion proteins blocked by the positive sera for antibodies against different subtypes of IAVs to react with the antigens was also determined using the blocking ELISA as described previously (27). Briefly, the ELISA plates were coated with the purified H9N2-NP (200 ng/well) at 4 °C overnight. Then, the positive and negative sera for anti-H9N2, H5N1, H7N9, H1N1, and H3N2 antibodies were added to the wells and incubated at room temperature for 1 h. After washing with PBST, the same amount of medium containing the nanobody-HRP fusion proteins (100 μl/well) was added to each well. After the absorbance at 450 nm values were obtained, the blocking rates were calculated using the following forum: (1 − [absorbance at 450 nm value of positive sera/absorbance at 450 nm value of negative sera]) × 100%. Then, the nanobody-HRP fusion protein being crossreacted with other subtypes of IAVs and with the largest blocking rate was selected for identification of recognizing epitopes and as a probe to develop the cELISA for detecting anti-IAV antibodies in different species.

### Identification of the epitope recognized by the nanobody-HRP fusion protein

To determine the epitope recognized by the selected nanobody-HRP fusion protein, a series of truncated and overlapping fragments from H9N2-NP were designed and expressed within the bacterial system. The H9N2-NP was first divided into two equal fragments and then, the two fragments were extended by 1/4 and 1/8 to the N-terminal region and C-terminal region, respectively (Fig. 4*A*). Based on the nucleotide sequences encoding H9N2-NP, primer pairs were designed for amplifying these fragments (Table S1). The expression and purification procedures of truncated fragments were the same as that for the full-length H9N2-NP. SDS-PAGE and Western blotting assays were used to analyze the expression and purification of these truncated fragments. Using these purified fragments as coating antigens, the antigenic domain recognized by the nanobody-HRP fusion protein was determined by direct ELISA.

To define the key amino acids involved in the interaction between the nanobody-HRP fusion protein and H9N2-NP more precisely, the 3D structures of homology modeling for H9N2-NP and the nanobody were generated by submitting the amino acid sequences of the two proteins to the AlphaFold2 server (28). The docking model of the interactions of the two proteins was then developed using the docking program on the server ClusPro (cluspro.bu.edu/home.php) (44). Interaction sites were analyzed using PyMOL (pymol.org/2/support.html) (45).

Subsequently, the predicted key amino acids were separately mutated to alanine (A) residues for H9N2-NP and the nanobody to identify the predicted results. The primers used for PCR amplification of the mutated fragments are listed in Table 1. The mutated H9N2-NP and nanobody-HRP fusion protein were expressed using the bacterial system and HEK-293T cells, respectively. Finally, direct ELISA using mutated H9N2-NP as the coating antigen and the nanobody-HRP fusion protein as the testing antibody was performed to confirm the predicted results.

### Amino acid alignment of the epitopes recognized by the nanobody-HRP fusion protein

To analyze the conservation of the epitope among the different subtype IAV strains, the amino acid sequences of the antigenic domains from different strains in GenBank were aligned using the Clustal W module of Lasergene 7.1 (DNASTAR, MegAlign). The GenBank accession numbers of different IAV strains were A/chicken/Hebei/0822/2007 (H9N2) (GQ373096.1), A/California/04/2009 (H1N1) (MN371617.1), A/swine/Guangxi/JG1/2014 (H3N2) (MF927881.1), A/HK/212/03 (H5N1) (AY575905.1), A/tree sparrow/Shanghai/01/2013 (H7N9) (KF609528.1), A/swine/Gunma/1/2012 (H1N2) (AB731586.1), A/Berkeley/1/68 (H2N2) (AY210104.1), A/canine/Florida/242/2003 (H3N8) (DQ124158.1), A/mallard/California/57871/2015 (H4N6) (KX351682.1), A/mallard/Sweden/100993/2008 (H7N7) (FJ803195.2), A/swan/Shandong/W4322/2020 (H10N4) (OM373303.1), A/mallard/Beijing/27-MA/2011 (H10N7) (KY688104.1), A/mallard/Korea/SH38-45/2010 (H13N2) (JX030409.1), and A/seagull/Chile/5775/2009 (H13N9) (KF772957.1).

### Development of the cELISA for detecting anti-IAV antibodies

Using the selected nanobody-HRP fusion protein as the probe, the cELISA for detecting anti-IAV antibodies in serum samples was designed as described previously (20). First, a checkerboard titration using direct ELISA was used to determine the optimal amount of coating antigen and dilution of the selected nanobody-HRP fusion protein. The amounts of coated H9N2-NP assessed were 50, 100, 200, and 400 ng/well, and the dilution ratios of the nanobody-HRP fusion assessed were 1:320, 1:640, 1:1280, 1:2000, 1:4000, and 1:6000 separately used for the direct ELISA. The optimized amount of antigen and dilution of nanobody-HRP fusion were determined when the pairs in the direct ELISA produced an absorbance at 450 nm value of ∼1.0.

Second, the dilutions of testing sera were optimized for the cELISA. The different dilutions (1:10, 1:20, 1:40, and 1:80) of five positive (P) and negative (N) sera for anti-IAV antibodies (each serum sample of H9N2, H1N1, H3N2, H5N1, and H7N9) were separately mixed with the optimized dilution of the nanobody-HRP fusion protein. After the optimized amount of H9N2-NP was coated into the plate, the mixtures were added to the plate and incubated for 1 h at 37 °C. Then, the TMB was added, and the absorbance at 450 nm values were read after the reaction was terminated. The optimal dilution of testing chicken sera was determined when the ratio of the positive and negative sera absorbance at 450 nm values (P/N) was the smallest.

Third, the incubation times between the mixture, the coated antigen, and the color reaction times after adding TMB were also optimized using the checkerboard assay. The incubation times of 20, 30, 40, 50, and 60 min and the color reaction times of 10 and 15 min were selected. Then, the two optimal times were determined when the P/N value was the smallest.

After the aforementioned conditions were optimized, the developed cELISA was performed as followed. The 96-well ELISA plates were coated with the optimized amount of H9N2-NP in PBS (0.1 M, pH 7.4) at 4 °C overnight. Then, the plates were washed and blocked with 300 μl/well blocking buffer (2.5% milk in PBST) at 37 °C for 1 h. After washing with PBST, 100 μl testing mixture containing the optimal dilutions of serum sample and nanobody-HRP fusion was added to wells and incubated for the optimal time at 37 °C. After washing three times, 100 μl TMB was added and incubated for the optimal time at room temperature, and then the absorbance at 450 nm values were measured after the reaction had been stopped by 3 M H_2_SO_4_.

### Validation of the cELISA

To calculate the PI of the cELISA, the 180 sera samples negative for anti-IAV antibodies from humans, pigs, and chicken were used. The cutoff value was set to the average PI of 180 negative sera plus three SDs to ensure 99% confidence that the negative sera were within this range.

The sensitivity of cELISA was evaluated by testing the different dilutions (1:10, 1:20, 1:40, 1:80, 1:160, 1:320, 1:640, 1:1280, and 1:2560) of 48 positive sera for anti-H9N2 (n = 16), H5N1 (n = 8), H7N9 (n = 8), H1N1 (n = 8), and H3N2 (n = 8) antibodies.

To determine the specificity of cELISA, a total of 181 clinical positive chicken sera against other avian viruses including NDV (n = 44), avian infectious bronchitis virus (n = 27), infectious bursal disease virus (n = 33), avian leukemia virus (n = 23), Marek’s disease virus (n = 21), and avian hepatitis E virus (n = 33), 128 positive pig sera against other swine viruses including porcine reproductive and respiratory syndrome virus (n = 23), porcine epidemic diarrhea virus (n = 27), porcine circovirus (n = 43), and pseudorabies virus (n = 23), and 69 positive human sera against other human viruses including hepatitis B virus (n = 26), severe acute respiratory syndrome coronavirus 2 (n = 15), and human adenovirus (n = 21) were used to test the developed cELISA.

The reproducibility of the developed cELISA was analyzed by testing five positive and negative sera to analyze the intra-assay and interassay variabilities. The CV was calculated using the PI values of different sera to evaluate the interassay variation (between plates) and the intra-assay variation (within a plate). Each serum sample was tested using three different plates to determine the interassay CV and three replicates within each plate to calculate the intra-assay CV.

The stability of the nanobody-HRP fusion and the developed cELISA were also analyzed. Briefly, the 96-well plates were coated with the purified recombinant H9N2-NP at 4 °C for 12 h. After washing with PBST and blocking with the blocking buffer, the plates were dried in a fume hood and vacuumed. Then, the plates and optimized dilutions of the selected nanobody-HRP fusion were stored at 4 °C. The direct ELISA and cELISA were separately performed as described previously after 0, 30, 60, 90, 120, 150, and 180 days to evaluate the stability.

### Analysis of the cELISA to detect anti-IAV antibodies in different species

To evaluate the developed cELISA for the detection of anti-IAV antibodies in the sera from different species, the 2155 sera from humans (n = 660), swine (n = 660), goats (n = 195), cattle (n = 28), rabbits (n = 44), cats (n = 44), pet dogs (n = 524), ducks (n = 98), and wild birds (n = 63) were both tested with the developed assay and an IDEXX IAV antibody ELISA kit (IDEXX).

### Comparisons of the analytical performance among the cELISA, commercial ELISA kits, and HI test

Reference methods for detection of anti-IAV antibody in laboratory and literature include HI test, agar gel immunodiffusion, virus neutralization test, IFA, and ELISA (5, 14). Among them, the HI assay is the serological “gold standard” method by OIE (38, 39). In addition, the ELISA is also widely used for testing anti-IAV antibody in the laboratory. Among these available commercial ELISA kits, the commercial IDEXX ELISA kit is currently the most stable and used for detection of anti-IAV antibodies. Therefore, the IDEXX ELISA kit and HI test were performed on a comparison of the analytical performance of the developed cELISA in this study.

The 185 clinical chicken sera and 91 sequential sera from 13 SPF chickens challenged with H9N2 AIV stock were simultaneously tested using the developed cELISA, a commercial IDEXX ELISA kit, and the HI test. Then, the coincidence rates were also calculated using Microsoft Excel’s CORREL function.

### Statistical analysis

Statistical analysis was performed using GraphPad Prism, version 5.0 (GraphPad Software, Inc). The *κ* index values were calculated to estimate the coincidence between the developed cELISA and the commercial ELISA kit (46). These calculations were performed using SPSS, version 20 (IBM Corp).

## Data availability

All data are provided within the article.

## Ethics

The animal experiments were performed in strict accordance with the recommendations in the Guide for the Care and Use of Laboratory Animals of the Northwest A&F University. The protocols used for animal and sera collections were approved by the Institutional Animal Care and Use Committee of Northwest A&F University (approval no. 20210028/09).

## Supporting information

This article contains supporting information.

## Conflict of interest

The authors declare that they have no conflicts of interest with the contents of this article.
