# Supplementary material for: A new nanobody-enzyme fusion protein–linked immunoassay for detecting antibodies against influenza A virus in different species
Source: J Biol Chem. 2022 Nov 17;298(12):102709. doi: 10.1016/j.jbc.2022.102709 (PMC9763686; doi:10.1016/j.jbc.2022.102709)
Supplement: Supporting information [file mmc1.docx]

**A new nanobody-enzyme fusion protein-linked immunoassay for detecting antibodies against influenza A virus in different species**

Pinpin Ji, ^1, ‡^ Kun Wang, ^1, ‡^ Lu Zhang, ^1^ Zhenda Yan, ^1^ Min Kong, ^1^ Xuwen Sun, ^1^ Qiang Zhang, ^1^ Ning Zhou, ^1^ Baoyuan Liu, ^1^ En-Min Zhou, ^1^ Yani Sun, ^1, *^ Xinjie Wang, ^2, *^ Qin Zhao ^1, *^

^1^ Department of Preventive Veterinary Medicine, College of Veterinary Medicine, Northwest A&F University, Yangling, Shaanxi, 712100, China.

^2^ Shenzhen Branch, Guangdong Laboratory of Lingnan Modern Agriculture, Genome Analysis Laboratory of the Ministry of Agriculture and Rural Affairs, Agricultural Genomics Institute at Shenzhen, Chinese Academy of Agricultural Sciences, Shenzhen, 518100, China.
^‡^ The two authors contributed equally to the paper.

^*^ Correspondences: sunyani@nwsuaf.edu.cn; wangxinjie@caas.cn; qinzhao_2004@nwsuaf.edu.cn.

**Supporting information**


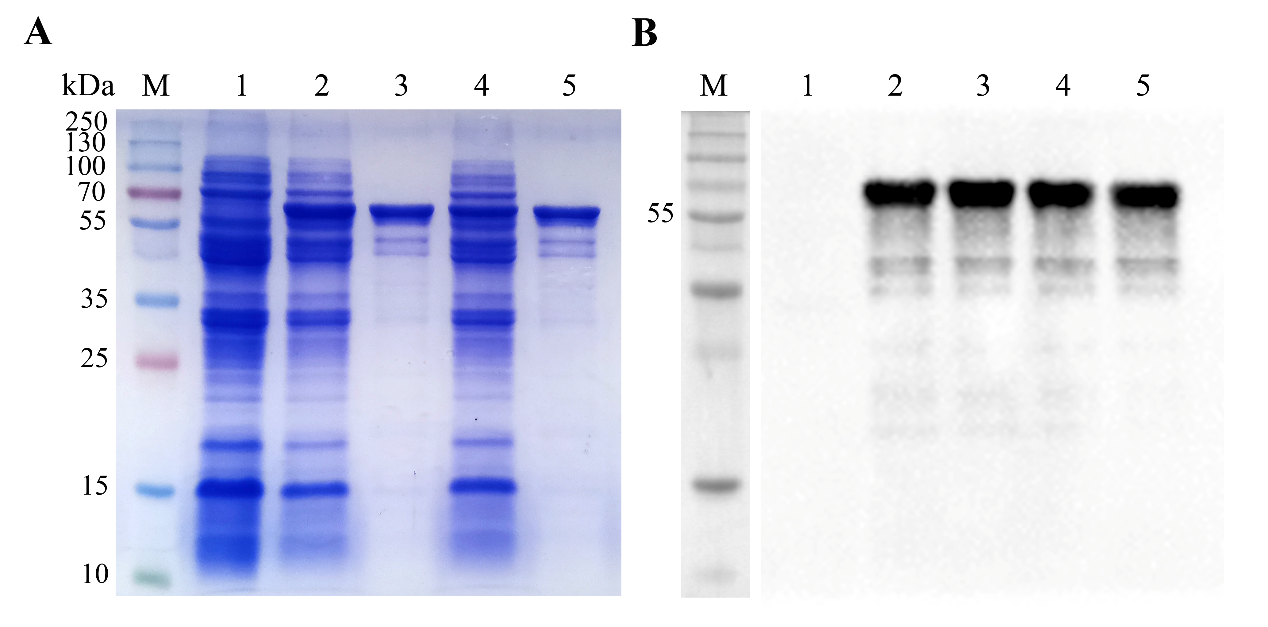


**Figure S1** Expression, purification, and identification of the recombinant H9N2‑NP protein. A, SDS‑PAGE analysis of protein expression. B, Antigenic analysis of the recombinant protein with western blotting using the positive chicken sera for anti‑H9N2 antibodies as the primary antibody. M, protein molecular markers; lane 1, pET‑28a vector control; lane 2, bacterial lysates of the recombinant protein; lane 3, inclusion body; lane 4, soluble protein; lane 5, purified protein.

**Table S1 Sequences of the primers used in this study.**

| **Primers** | **sequences（5’-3’）** | **Usage** |
| --- | --- | --- |
| H9N2-NP-F | GATGAATTCATGGCGTCTCAAGGCACCAA | pET28a-H9N2-NP |
| H9N2-NP-R | GACCTCGAGTCAATTGTCATACTCCTCTGCATT |  |
| CALL001 | GTCCTGGCTGCTCTTCTACAAGG | Overlap-VHH |
| CALL002 | GGTACGTGCTGTTGAACTGTTCC |  |
| VHH-FOR (*Pst* I) | CAGGTGCAGCTGCAGGAGTCTGGGGGAGR |  |
| VHH-REV (Not I) | CTAGTGCGGCCGCTGAGGAGACGGTGACCTGGGT |  |
| H9N2-NP-F187 | GATGAATTCATAGAGAGAATGGTA | Truncated fragments from pET28a-H9N2-NP |
| H9N2-NP-F373 | GATGAATTCAATGGAGAGGATGCAACT |  |
| H9N2-NP-R1122 | GACCTCGAGTCACATTGCTTCCATGTTCTC |  |
| H9N2-NP-R1308 | GACCTCGAGTCATCTACCCTCAGTATT |  |
| H9N2-NP-R1215 | GACCTCGAGTCACTGTCCTGCAGATGC |  |
| H9N2-NP-R1152 | GACCTCGAGTCATCTACTTCTCAGTTC |  |
| H9N2-NP-R1167 | GACCTCGAGTCATCTTATAGCCCAATA |  |
| H9N2-NP-R1182 | GACCTCGAGTCACCCTCCGCTTCTGGT |  |
| H9N2-NP-R^388-389M^ | TTCTGGTCGCCGCAGCCCAATATCTACT | pET28a-H9N2-NP^388-389M^ |
| H9N2-NP-F2^388-389M^ | ATTGGGCTGCGGCGACCAGAAGCGGAG |  |
| H9N2-NP-R^385-389M^ | CTTCTGGTCGCCGCCGCCGCCGCTCTACTTCTCAG | pET28a-H9N2-NP^385-389M^ |
| H9N2-NP-F2^385-389M^ | GCGGCGGCGGCGGCGACCAGAAGCGGA |  |
| H9N2-NP-R1^3M^ | CTGGTTCTTATCGCCGCCGCTCTACTTCTCAGTTCAAGAGTATTGGAGTCCATTGCTTCCATGTTCTCATTTGAAGCAATTTGAACCCCTCTAGTGGATAGCTGCGCCGCCGCGACCATTCTTGTC | pET28a-H9N2-NP^3M^ |
| H9N2-NP-F2^3M^ | CTGAGAAGTAGAGCGGCGGCGATAAGAACCAGA |  |
| H9N2-NP-R2-1^3M^ | CGTTGCCTTTTCGTCCGAGAGCTCGAAGACTCCCCGTCCCTGGAATGACACATCTTCTGGTCTGGCACTCGCCGCCGCCGCCGCGATTTCAGTCCT |  |
| H9N2-NP-R2-2^3M^ | CCGCTCGAGTCAATTGTCATACTCCTCTGCATTGTCTCCGAAGAAATAAGATCCTTCATTATTCATGTCAAAGGAAGGCACGATCGGGTTCGTTGCCTTTTCGTCCGAGAGCTCG |  |
| Nb5^W95A, T96A, K100A^-HRP-F | CGCGGATCCGAGTCTGGGGGAGGC | H9N2-NP- Nb5^W95A, T96A, K100A^-HRP |
| Nb5^W95A, T96A, K100A^-HRP-R | CGGGGTACCTGAGGAGACGGTGACCTGGGTCCCCTGGCCCCGAGACGCATATCCAGACGCCGCGCCTCT |  |

^a^Restriction sites are underlined.

| **Round of banning** | **Input phage**  **(pfu/well)** | **P output phage**  **(pfu/well)** | **N output phage**  **(pfu/well)** | **Recovery**  **(P/input)** | **P/N** |
| --- | --- | --- | --- | --- | --- |
| 1^st^ round | 5.0 × 10^10^ | 5.8 × 10^6^ | 9.1 × 10^6^ | 1.2 × 10^-4^ | 0.64 |
| 2^nd^ round | 5.0 × 10^10^ | 8.7 × 10^7^ | 6.0 × 10^4^ | 1.7 × 10^-3^ | 1.5 × 10^3^ |
| 3^rd^ round | 5.0 × 10^10^ | 5.2 × 10^7^ | 9.0 × 10^2^ | 1.0 × 10^-3^ | 5.8 × 10^4^ |

**Table S2 Three-round panning of phage particles against the H9N2-NP protein.**

**Table S3 Determination of the optimal quantity of H9N2-NP protein as the coating antigen and dilution of H9N2-Nb5-HRP fusions in the medium using the direct ELISA.**

| Amounts of H9N2-NP proteins (ng/well) | Different dilutions of H9N2-Nb5-HRP fusion in the medium | | | | | |
| --- | --- | --- | --- | --- | --- | --- |
|  | 1:320 | 1:640 | 1:1280 | 1:2000 | 1:4000 | 1:6000 |
| 50 | 1.6 | 1.3 | 1.2 | 0.77 | 0.64 | 0.55 |
| 100 | 1.9 | 1.9 | 2.0 | 1.5 | 1.1 | 0.88 |
| 200 | 2.9 | 2.7 | 2.4 | 1.8 | 1.3 | 1.2 |
| 400 | 3.0 | 2.8 | 2.6 | 1.9 | 1.5 | 1.2 |

**Table S4 The optimal dilution of tested sera for the developed cELISA.**

| Anti-IAV antibodies | Sera type | 1:10 | 1:20 | 1:40 | 1:80 |
| --- | --- | --- | --- | --- | --- |
| H9N2 | Positive | 0.18 | 0.20 | 0.23 | 0.40 |
|  | Negative | 1.5 | 1.4 | 1.4 | 1.4 |
|  | P/N | 0.12 | 0.14 | 0.16 | 0.29 |
| H1N1 | Positive | 0.47 | 0.55 | 0.56 | 0.75 |
|  | Negative | 1.4 | 1.4 | 1.4 | 1.4 |
|  | P/N | 0.34 | 0.39 | 0.40 | 0.54 |
| H3N2 | Positive | 0.13 | 0.12 | 0.17 | 0.31 |
|  | Negative | 1.4 | 1.5 | 1.3 | 1.4 |
|  | P/N | 0.093 | 0.080 | 0.13 | 0.22 |
| H5N1 | Positive | 0.16 | 0.22 | 0.34 | 0.64 |
|  | Negative | 1.4 | 1.5 | 1.4 | 1.3 |
|  | P/N | 0.11 | 0.15 | 0.24 | 0.49 |
| H7N9 | Positive | 0.13 | 0.17 | 0.39 | 0.58 |
|  | Negative | 1.4 | 1.4 | 1.4 | 1.3 |
|  | P/N | 0.093 | 0.12 | 0.28 | 0.41 |

**Table S5 Optimized incubation time of the mixture containing chicken sera and H9N2-Nb5-HRP fusions incubated with the antigen and optimal time for the colorimetric reactions following the addition of TMB using a checkerboard assay with the developed cELISA.**

| Time of color reaction (min) | Sera type | Incubation time of the mixture with the coated antigen (min) | | | | |
| --- | --- | --- | --- | --- | --- | --- |
|  |  | 20 | 30 | 40 | 50 | 60 |
| 10 | Positive | 0.28 | 0.36 | 0.49 | 0.26 | 0.26 |
|  | Negative | 1.0 | 1.2 | 1.5 | 1.2 | 1.2 |
|  | P/N | 0.28 | 0.30 | 0.33 | 0.22 | 0.22 |
|  | Positive | 0.21 | 0.19 | 0.21 | 0.18 | 0.14 |
|  | Negative | 1.0 | 1.2 | 1.3 | 1.1 | 1.2 |
|  | P/N | 0.21 | 0.16 | 0.13 | 0.16 | 0.122 |
|  | Positive | 0.20 | 0.26 | 0.21 | 0.18 | 0.16 |
|  | Negative | 1.0 | 1.2 | 1.3 | 1.1 | 1.2 |
|  | P/N | 0.20 | 0.22 | 0.16 | 0.16 | 0.13 |
| 15 | Positive | 0.86 | 0.77 | 0.84 | 0.93 | 0.74 |
|  | Negative | 1.1 | 1.1 | 1.3 | 1.4 | 1.2 |
|  | P/N | 0.78 | 0.70 | 0.65 | 0.66 | 0.62 |
|  | Positive | 0.43 | 0.39 | 0.40 | 0.45 | 0.38 |
|  | Negative | 1.0 | 1.1 | 1.2 | 1.3 | 1.2 |
|  | P/N | 0.43 | 0.33 | 0.33 | 0.35 | 0.32 |
|  | Positive | 0.19 | 0.18 | 0.19 | 0.19 | 0.15 |
|  | Negative | 1.0 | 1.1 | 1.2 | 1.3 | 1.2 |
|  | P/N | 0.19 | 0.16 | 0.16 | 0.15 | 0.13 |

**Table S6 Reproducibility of the cELISA determined intra and inter-assay CV % values.**

| **Type of precision** | **CV % value range of 5 serum samples** | **Median value** |
| --- | --- | --- |
| Intra assay precision (CV%) | 3.1-7.0 | 5.1 |
| Inter assay precision (CV%) | 4.7-12.7 | 8.7 |

Intra-assay precision: Determined from three repetitions (well-to-well) of 5 serum samples in the same method.

Inter-assay precision: Determined from three repetitions (plate-to-plate) at different time.

cELISA, competitive ELISA; CV, coefficient of variation.
